# Study-related demands, resources and curriculum from students’ perspective: results of a qualitative descriptive study among students in nursing and healthcare in Germany

**DOI:** 10.1186/s12912-025-03692-8

**Published:** 2025-08-11

**Authors:** Ivonne-Nadine Jürgensen, Annike Morgane Nock, Peter Koch, Albert Nienhaus, Corinna-Petersen Ewert

**Affiliations:** 1 Department of Nursing and Management, Faculty of Business and Social Science, University of Applied Sciences Hamburg, 20099 Hamburg, Germany; 2 Competence Center for Epidemiology and Health Services Research for Healthcare Professionals (CVcare), Institute for Health Services Research in Dermatology and Nursing (IVDP), University Medical Center Hamburg-Eppendorf, 20246 Hamburg, Germany; 3 Department for Occupational Medicine, Hazardous Substances and Health Sciences (AGG), German Social Accident Insurance for the Health and Welfare Services (BGW), Hamburg, Germany

**Keywords:** Nursing students, Academic stress, Resources, Health promotion, Students’ perspective

## Abstract

**Background:**

Health-related studies are associated with numerous academic and clinical demands that can lead to stress and mental strain. Therefore, university interventions to promote health and well-being are important. However, the academic sources of stress and resources for the group of nursing and healthcare students have not yet been explored in Germany. The aim of this study is therefore to investigate these factors from the students’ perspective.

**Methods:**

A descriptive qualitative study was conducted. Findings are based on interviews with students from the Department of Nursing and Management in Germany, carried out between October 2022 and January 2023. The interviews were audio‑recorded, transcribed, and analyzed using qualitative content analysis. Results are presented in a descriptive summary.

**Results:**

Participants reported performance demands, time pressure, group tasks, peer relationships, as well as organizational factors and ergonomic conditions within learning environments as primary stressors in their studies. Essential resources highlighted by the students included opportunities for personal development, physical activity, peer support, and individual competencies. Health was conceptualized by the interviewees as multidimensional, encompassing mental, physical, and social aspects. Integrate a health module into the curriculum was met with ambivalence among participants; on the one hand, it was perceived as an opportunity to enhance awareness of health and self-care, while on the other hand, there was concern regarding potential overload through mandatory obligations. However, participants agreed that a balanced approach, incorporating structured offerings alongside room for individual autonomy, is essential.

**Conclusions:**

In summary, this study identified study-related stress factors and their impact on mental well-being from the students’ perspective. The stressors reported by our students largely correspond to those identified in international studies involving nursing students in other countries. The current study is limited by the small sample. However, the findings highlight the importance to create healthy study conditions. Findings are particularly relevant for universities and educators in Germany, who would like to become more aware of the study-related stressors of nursing and healthcare students and discuss suitable preventive measures. Further quantitative research should examine the academic demands and their impact on students’ mental health. A nationwide, representative study would be a valuable next step.

**Supplementary Information:**

The online version contains supplementary material available at 10.1186/s12912-025-03692-8.

## Introduction

In this paper, we present the findings of a descriptive qualitative study that we conducted with students from the Department of Nursing & Management at a University of Applied Sciences in Germany (Hamburg). Our study is the first in Germany to qualitatively examine, from the students’ perspective, the stressors associated with studying in nursing and healthcare professions programs. The aim of the study was to examine which demands of studying are perceived as stressful by our students and how these affect their subjective health. We were also interested in what resources students use to cope with the demands and how they define health.

### Background our study

In Germany, the academization of the health professions—particularly nursing—is progressing [1, 2]. As part of this process, the Nursing Professions Act (2020) (Pflegeberufegesetz) [1] has enabled a primary qualifying higher education programme in nursing. The aim of academic nursing education is to improve the quality of healthcare and to prepare future professionals for the increasingly complex demands of patient care [1, 2]. In the course of these changes, experienced professionals also play a crucial role in the academic advancement of the German health sector [3]. Nursing degree programmes in Germany combine university-based education with clinical practice [1], resulting in a highly structured curriculum with limited temporal flexibility and little room for autonomous learning [1, 4]. These programmes began in 2020 [1]. However, there is still limited knowledge about students’ stressors, health status, and health promotion needs [5]. To date, only two studies have been published: Zimmermann et al. [6] examined nursing students’ motivation and satisfaction in light of the new legislation, while Großmann et al. [7] conducted a nationwide longitudinal study focusing on study motivation, curriculum structure, clinical learning environments, and overall programme evaluation [7]. These developments in nursing education are embedded within broader dynamics affecting students in higher education more generally: Entering university represents a formative phase in young adults’ lives, marked by diverse challenges [8, 9]. Arnett [10] refers to this life stage between the ages of 18 and 25 as “emerging adulthood,” characterized by the pursuit of autonomy, instability, and increased vulnerability to stressors and risky behavior [10]. During this sensitive periode, students encounter a higher education system that has undergone significant transformation due to profound structural changes, notably the Bologna Process—a Europe-wide agreement that has compressed and modularized study content [11]. This transformation has contributed to a wide range of academic demands that increasingly lead to stress, exhaustion, and mental strain among students in Germany [11–16]. A recent meta-analysis based on 56 studies, found that nearly one in five students in Germany shows symptoms of depression [17]. This underscores the urgent need for health-promoting interventions in higher education [15, 17]. The World Health Organization (WHO) highlights the central role of universities as sites for health promotion [18]. University-based initiatives can enhance well-being, support academic success, and reduce health inequalities [18, 19]. From a systems theory perspective, the relevance of such interventions becomes even clearer [20]. According to Becker (2006), health is determined by how individuals manage internal and external demands using available resources. Demands may stem from the environment (e.g., academic workload) or from within the individual (e.g., personal aspirations), while resources may include internal factors (e.g., competencies) or external supports (e.g., social networks). Based on this model, Becker calls for interventions tailored to the target group and focused on promoting subjective health needs [20]. The following section presents empirical findings from Germany regarding study-specific demands and their impact on students’ health:

### Study-related demands and health

Grützmacher et al. [13] show that approximately one quarter of students experience high study-related stress, with women (29,2%) being significantly more affected than men (21,4%) [13]. Herbst et al. [12] report that particularly university and bachelor’s degree students experience especially high levels of stress [12]. Current data confirm that 44% of students in Germany feel chronically stressed, primarily due to exam pressure and a high workload [15]. Chronic stress refers to a prolonged state of overload in which existing coping strategies are no longer sufficient to manage the demands [21]. Chronic stress can lead to psychological symptoms such as inner restlessness, tension, and worry, as well as physical complaints including pain, cardiovascular problems, or gastrointestinal disorders [21]. An online survey among students at the Healthy Campus Mainz [16] found that 41% of participants suffer from depressive disorders, and 36% report feelings of nervousness and worry [16]. Representative data from a large German health insurance (Techniker Krankenkasse) [15] shows that students who face multiple stressors, such as exam pressure and complex learning content, report exhaustion particularly frequently [15]. Exhaustion is considered a core symptom of burnout syndrome [22]. Wörfel [23] identified experiences of overwhelm, time pressure, and the difficult reconciliation of study and private life as key health-impairing factors related to studying [23]. Hübner et al. [24] demonstrate that particularly high time and cognitive demands related to studies are significantly associated with exhaustion [24]. The combination of study-specific stressors, high demands, and limited scope for action demonstrably impairs the health and quality of life of students [23, 24]. Within the empirically validated Study Demands-Resources Framework, time pressure, exam stress, and high workload are described as stressful study conditions that can increase the risk of psychological distress and burnout [25].

### Study-related resources and health

While certain study-specific demands can adversely affect students’ health and well-being, university education simultaneously offers a variety of health-related resources [23]. Health-related resources are factors that contribute to maintaining health by helping to manage demands and reduce stress [26]. A central resource in everyday student life is social support [27, 28]. Students benefit from functional support among peers [27, 28], while professors play an important role through their accessibility and constructive feedback [24]. These forms of support are associated with higher subjective quality of life [24, 27] as well as improved mental well-being among students [28]. Social support is understood as a qualitative characteristic of social relationships, manifesting in instrumental, emotional, or informational assistance, and generally promotes health or buffers stress and other burdens [29]. Poots and Cassidy [30] also found that greater social support was positively associated with students’ well-being and lower stress levels [30]. In this context, Blau et al. [31] demonstrated that social connectedness is a key predictor of students’ life satisfaction and should be strengthened through targeted university support services [31]. Moreover, Kirsch et al. [32] found that greater choice and individual autonomy within the study programme were associated with better self-rated health and higher life satisfaction [32]. Additionally, sufficient time availability emerged as an important resource for students and was associated with higher quality of life [27]. Keller and Semmer [33] emphasized that autonomy is central to achieving work goals, can reduce stress, and is positively associated with students’ motivation, well-being, and health [33]. Moreover, overall satisfaction with study conditions appears to benefit students’ health; Lehnchen et al. [34] document that higher satisfaction with study conditions was significantly associated with lower mental distress [34]. Within the empirically validated Study Demands-Resources Framework, the anticipated qualification potential as well as social support from fellow students and instructors are described as key study-related resources that can positively influence students’ engagement and health [25].

### Students in health-related programmes

Nursing students experience higher overall stress levels than other university students. International studies show that they must cope not only with academic stressors [35] but also with the demands of the clinical learning environment [36]. This environment entails early responsibility for patient care and substantial emotional and cognitive demands, while supportive services are often insufficient [37–39]. Consequently, many students struggle to prioritize their own health and self-care [40]. Together, these factors impose additional burdens, contributing to stress and adversely affecting mental health and well-being [35–40].

### Summary

Students face a variety of demands throughout their studies, some of which pose a burden to their health. However, no study in Germany has been examined nursing and healthcare students’ perspectives of study-related demands, resources, and factors that determine their health. The novelty and significance of this study is to address this research gap. The aim of the present study is therefore to identify which demands students perceive as particularly stressful and which resources they use to cope with these challenges. Gaining such insights appears especially significant in the context of the ongoing academization of nursing in Germany [1, 2] and the limited knowledge about this group of students in Germany [5–7].

### Theoretical framework

For our study, we used the Study Demands-Resources Framework [25]. This framework served as a guiding structure for our research questions and the development of the interview guide. To capture the relevant dimensions, the interview guide included the following questions, for example *Which conditions and challenges of your studies affect your health? What resources contribute to maintaining and promoting your health?* These questions serve to examine the students’ perspectives by dividing them into two main topics: on the one hand, the stresses and challenges (demands) that can have a negative impact on health, and on the other hand, the supporting factors and resources that contribute to maintaining and promoting health. For the data analysis, the responses were categorized according to these primary areas. In this way, the framework not only facilitates the organization of data collection but also improves the analytical process.

### Study aims

The aim of this study is to examine.

- how students define health.
- which stress factors they experience during their studies.
- which supporting factors they find helpful and.
- their attitudes towards the integration of health promotion into the curriculum.

Based on this, the following research questions have been formulated:

1. **How do students define and understand health?**

(This question seeks to explore how students conceptualize health. Understanding their view of health is crucial for designing health‑promotion interventions that resonate with their personal perspectives and competencies.)

(2) **What study-related demands and resources do students perceive**,** and how do these demands and resources influence their health and well-being?**

(This question seeks to identify which study‑related demands students view as risk factors, which they regard as resources for their health and well‑being, and how these factors influence their overall health.)

(3) **What attitudes and ideas do students perceive regarding the integration of health promotion into the curriculum?**

(Students face a range of practical and academic challenges during their studies, and their perspectives can provide valuable insights into whether and how health-promoting content can be integrated into the curriculum.)

## Methods

### Qualitative descriptive study

To answer our research questions, we chose a qualitative descriptive study based on Sandelowski [41]. This approach allows for a collection of perspectives and perceptions of events and phenomena that is as close to everyday experience as possible, without heavy interpretation or theoretical abstraction. Qualitative descriptions remain closely tied to the data collected, as well as to the participants’ language and lived experiences, enabling a perspective that is as natural and everyday as possible [41]. The qualitative descriptive approach results in a summary of the findings in everyday, factual language [41, 42]. In the context of our research interests, this approach seems appropriate for capturing the subjective perspectives of our students—both regarding health-related challenges during their studies and the supportive factors that help them to manage these challenges in a health-promoting way. Additionally, we are interested in how students perceive the integration of health promotion into the curriculum and what attitudes they share regarding it. Another advantage of the chosen methodological approach is the ability to present students’ statements in their own language, without distorting them through abstract theoretical concepts. The goal is not to develop theoretical explanatory models, but rather to provide the most comprehensive and everyday-oriented representation of our students’ perspectives [41, 42]. Our intention is not to develop theoretical explanatory models, but rather to provide a comprehensive and everyday-oriented representation of our students’ perspectives.

### Reflection

The qualitative descriptive approach allowed me to select a suitable evaluation strategy that fit my research question. At the same time, I was able to select my interview partners in such a way that they contributed to answering the research questions. Furthermore, the method allowed me to collect data at a low threshold, which facilitated access for the participants and enabled me to collect data that reflected the actual experiences and perspectives of the interviewees as accurately as possible. As a junior researcher, this method provided me an ideal opportunity to deepen my skills in qualitative research and gain valuable practical experience. I was aware that my professional proximity to the university could influence my assumptions. Throughout the research process, I continuously reflected on this by taking detailed notes and creating case summaries of the interview participants. In addition, I regularly discussed my reflections and the developed categories in the research team as well as with my colleague (AMN) to receive feedback, broaden my perspectives, incorporate different points of view and minimize potential biases.

### Participants and setting

The study was conducted at a German university. Participants were students from the Department of Nursing and Management. Eligibility for participation in the interviews was limited to students who had reached the age of 18 (the age of majority under German law), were proficient in the German language—since the interviews were conducted in German—and were enrolled in the Nursing (Bachelor’s or Master’s degree) or Interdisciplinary Healthcare and Management (Bachelor) programs. Students under the age of 18 were excluded from participation, as this age threshold corresponds to the legal capacity to provide informed consent independently. Furthermore, students with insufficient German language skills, as well as those who were on leave or engaged in practical semesters during the survey period, were excluded, as they were not actively involved in the regular academic program and thus did not experience typical student life during this time.

### Data collection

#### Interview guide

The contextual knowledge from the existing research (see introduction) and the three research questions formed our basis for the formulation of the interview questions, for example: *What demands and challenges in your studies affect your health?* as well as for structuring the topic blocks in the interview guide, for example: Study demands. The questions for the interview guide were selected using the spss method according Helfferich [43] (Appendix 1). Helfferich’s spss method [43] is a structured approach for developing interview guidelines in qualitative research. **spss** stands for:

- →(**s**) Collecting: All potential questions are collected in an open brainstorming session - without filters or evaluation.
- →(**p**) Review: The collected questions are critically examined for their suitability, openness and relevance to the research objective.
- →(**s**) Sorting: Suitable questions are organized thematically.
- →(**s**) Subsuming: The sorted questions are summarized under superordinate narrative stimuli that promote the interview flow.

The aim of the method is to create a flexible but systematically structured guide. The guide was developed and evaluated by three research colleagues (INJ, AMN, CPE).

#### Data collection process

The recruitment of participants took place via two channels: first, students were approached personally during lectures and invited to participate in an interview on the topic of “Studies and Health.” Second, an invitation to participate was sent to all students via email. A total of twenty interviews were planned, guided by both practical time constraints and existing methodological literature [44]. Moreover, data saturation was deemed attained when no further insights relevant to the research questions emerged [44]. To substantiate this judgment, the researcher prepared a concise case summary for each participant during the initial text‑analysis phase and regularly discussed the material with a research colleague (AMN). Students who expressed interest in participating contacted the researcher independently, after which an interview appointment was scheduled. Prior to conducting the interviews, participants were verbally informed about the study’s aims and procedures. It was explicitly emphasized that participation was voluntary and had no impact on any academic performance or evaluations. The interviews were conducted in German, both face-to-face and digitally. The face-to-face interviews took place in publicly accessible rooms on campus. Before each interview, written informed consent was obtained. The digital interviews were recorded using appropriate software. In total, eight interviews were conducted in person and twelve digitally. Interview durations ranged between 15 and 35 min. The interviews were conducted between October 2022 and January 2023. For the present publication, relevant findings and selected quotes were translated from German into English using the online translation tool DeepL. The translations were subsequently reviewed by a colleague (AMN). No member-checking was conducted as part of this study.

#### Interview transcription

The interviews were transcribed via computer-assisted software *f4x* (audiotranskription | dr. dresing & pehl GmbH, Marburg, Germany). The transcripts were subsequently checked for completeness and processed via transcription rules [45]. Any unclear passages were revisited and clarified by cross-checking with the original audio recordings. The finalized transcripts served as the primary data source for subsequent qualitative analysis.

### Data analysis

The aim of the data analysis was to identify central themes within the data, condense it into key aspects and generate a descriptive summary aligned with the research questions [41]. To achieve this, a content-structuring qualitative content analysis following Kuckartz [46] was applied, as it follows a systematic, category-based analysis of the data [46]. Kuckartz’s approach is based on a clearly defined, seven-step procedure, which ensures a transparent, replicable, and structured process of category development and data analysis [46] (1) initiating text work, (2) development main categories, (3) coding the entire data material using the main categories, (4) compilation of all text segments assigned to the same main category, (5) inductively development subcategories, (6) recoding of the entire material using the refined category system and (7) conducting a analysis using the main- and subcategories. This seven-stage process was fully completed:

1. Initiating text work: Each transcribed interview was printed out. The interview transcripts were read several times making sense of the data as a whole and edited manually, e.g. by highlighting relevant text passages. At the same time, short case summaries were written.
2. Development main categories: Five main categories were deductively derived from the interview guide: health, study requirements, resources, curriculum, and health promotion. Content that could not be clearly assigned was temporarily placed under the category “Miscellaneous.”
3. Coding the entire data material using the main categories: Coding was performed using the QDA software f4analyse (audiotranskription | dr. dresing & pehl GmbH, Marburg, Germany). First, all interview transcripts were transferred to the software. To enhance intersubjective comprehensibility, the first interview was independently coded by two researchers (INJ & AMN); discrepancies were resolved through consensus. Subsequent interviews were coded by INJ; uncertainties were discussed jointly with AMN.
4. Compilation of all text segments assigned to the same main category: A dedicated module was created in f4analyse for each main category, collecting all relevant text segments.
5. Inductively development subcategories: Within each main category, subcategories were developed inductively, adhering to the principle “as concise as possible, as differentiated as necessary,” and saved in f4analyse as separate subcategories within the respective main category.
6. Finalization of the category system and recoding: All interview transcripts were reviewed a second time in f4analyse and fully coded using the finalized system of main categories and subcategories.

Analytical interim step: After coding the entire data set, a thematic summary was created in tabular form (rows representing interviewees, columns representing categories). All coded text passages and references were transferred into the table. A second document contained a content-based condensation of the text segments focusing on research-relevant aspects. This condensation facilitated the subsequent theme-oriented presentation.

7. Descriptive analysis according to main categories: Themes and subthemes were summarized descriptively and illustrated using representative quotations.

The results are presented below in line with our research questions.

## Results

### Details of the interviewees

As shown in Table 1, twenty students from the Department of Nursing and Management participated in this study, including 13 females and 7 males. The median age of the participants was 27 years, with an age range of 22–64 years. With respect to marital status, ten participants were single, four were in a relationship, and five were married. Seventeen participants had completed vocational training or degrees in healthcare professions, such as nursing or therapy. The participants’ professional experience varied: nine had between 0 and 5 years, five had between 6 and 10 years, and six had over 10 years of experience. Eighteen participants held a part-time job while studying. In terms of financial support, nine participants received assistance from parents or government funding.

**Table 1:** Characteristics of the interviewees (*n* = 20)

Median age (range)	27 (22–64)
**Gender**	*n*
Female	13
Male	7
**Marital status**	**n**
Single	10
In Relationship	4
Married	5
**Vocational training/study in a health profession**	**n**
Yes	17
Nursing Profession Therapy Profession	7 10
**Professional experience (in years)**	**n**
0–5	9
6–10	5
> 10	6
**Part-time job during studies?**	**n**
Yes	18
**Financial support?** (e.g., from parents or government funding)	**n**
Yes	9

The major themes and their subthemes are depicted in Table 2.

**Table 2:** Overview of the main categories and their subcategories (Coding Frame)

Main category (from interview guide)	Subcategory (from data material)
Definition of Health	o Mental health o Physical health
Study-related Demands, Resources and Health	**Demands** o Performance Demands & Time Pressure o Group Work and Peer Relationships o Organization and Learning Environment **Resources** o Anticipated Potential for Personal Development o Physical Activity and Social Support o Personal Resources
Curriculum and Health Promotion	o Individual and Voluntary o Mental Health and Wellbeing

### How do students define and understand health?

Students do not understand health as a one-dimensional or exclusively biomedical concept, but rather as a complex interaction of two different dimensions: physical and mental. This interdependence makes it clear that health should always be considered in the context of the individual reality of life. At the centre of this is the subjective perception of well-being, which shows that the understanding of health is significantly shaped by personal experiences and everyday life.

### Mental health

The emphasis on mental well-being and personal skills shows a resource-orientated perspective. For the students, health does not only mean the absence of mental illness, but also the ability to actively and reflectively face the challenges of everyday life. Mental health is seen as a prerequisite for life satisfaction and self-efficacy - a perspective that recognizes resilience and mental stability as central aspects of health.

*“Health is actually being happy. Being content with your body and yourself. In addition*,* most importantly*,* being mentally healthy and stable”* (E7: paragraph 18). *“The ability to do everything I envision*,* without having limitations or impairments. In addition*,* yes*,* having both mental and physical balance and being able to live a pain-free everyday life*,* so to speak”* (E2: paragraph 22).

### Physical health

The physical level is primarily addressed in connection with the ability to act and autonomy. Health is understood as physical integrity, but also as the ability to actively participate in everyday life. This connection between physical integrity and social agency points to a functional understanding of health that is closely linked to self-determination and social inclusion.

*“Health means well-being to me*,* the absence of illness*,* and motivation*,* I would say*,* plays a role for me. These are the three points where I would then say*,* I am healthy”* (E13: paragraph 20). *“Health means having energy to me*,* feeling comfortable with my social environment*,* and in my body. However*,* also achieving what I set out to do*,* meaning my personal goals”* (E18: paragraph 22).

The students’ understanding of health corresponds to Faltermaier’s subjective concept of health, according to which health is understood as a phenomenon embedded in everyday life that is characterized and defined by individual resources, biographical experiences and coping strategies [47].

### What study-related demands and resources do students perceive, and how do these demands and resources influence their health and well-being?

### Study-related demands (Fig. 1)

#### Performance demands & time pressure

Coping with academic demands alongside professional and personal commitments is a considerable burden for many students. They often report increased stress, a subjective lack of time and the feeling of being overwhelmed - especially at the beginning of their studies. Poorly structured examination formats and a lack of transparency in the organization of performance assessments further increase this burden. Chronic lack of time has a particularly negative impact on subjective well-being and mental health.

*“The most significant impact on my health is the pressure to complete tasks by certain deadlines*,* alongside family and work commitments. This is the biggest challenge and burden during my studies”* (E1: paragraph 37). *“Yes*,* indeed. The organizational aspect is a major factor that becomes a stressor affecting my health”* (E12: paragraph 32). *“Ever since I started my studies*,* my stress levels have increased enormously. Juggling so many different things. The time-related stress has increased tremendously”* (E17: paragraph 33).

The increasing demands on performance and the resulting lack of time mean that students are increasingly neglecting health-promoting activities. In particular, physical exercise and concern for personal well-being are often suppressed, especially during intensive examination phases. Another important issue is the interaction between stress, procrastination and health: procrastination may bring short-term relief, but in the long term it leads to more stress and puts an additional strain on health. This form of self-denial not only manifests itself in short-term symptoms such as exhaustion and tiredness but can also lead to long-term impairment of physical and mental health.

*“When studies take up a lot of time*,* there’s less time for physical exercise. In addition*,* it obviously stresses you out a lot during intense periods*,* which makes you very tired”* (E5: paragraph 32). *“I do not know if it is just me*,* but when an exam period or a stressful time*,* like with an exam*,* is coming up*,* my thoughts truly focus on that. It is a mix of working intensely and procrastinating*,* which somehow spirals into stress*,* and then I often do not pay much attention to health-related things”* (E13: paragraph 32).

#### Group work & peer relationships

Another stress factor is group work, which is seen as conducive to learning but at the same time organizationally challenging. Coordination in heterogeneous groups, different working styles and the time availability of group members often lead to conflicts and additional coordination efforts. This additional effort is described by many as time-consuming, labour-intensive and emotionally stressful.

*“Oh*,* what was a bit exhausting were the many group projects. Having to coordinate a lot with others*,* which just led to scheduling difficulties*,* and not all personalities necessarily matching”* (E4: paragraph 48). *“Stress levels have increased*,* many project groups. It is always different*,* from semester to semester. Not every semester has been the same. However*,* these project groups definitely cause considerable time stress. In addition*,* coordinating with different groups always requires one to mentally stay on top of things. In addition*,* that can certainly lead to a lot of emotional stress”* (E3: paragraph 36). *“Oh*,* the group projects! The group projects definitely affect my health in their entirety and the intensity of the workload”* (E12: paragraph 30).

#### Organization & learning environment

The organizational structure of the degree programme and the design of the learning environment have a significant influence on students’ stress levels. Respondents criticized in particular last-minute changes to courses, unclear processes, lack of availability of lecturers and inadequately communicated timetables. These factors increase the need for students to organize themselves and often lead to planning uncertainties.

*“What is stressful is the organization*,* whether it is in person or online. Which professor is more available and answers questions […] and which professor cannot be reached. The professor situation*,* meaning the teaching situation*,* that is not fully organized and becomes unreliable at times owing to the guidance from professors”* (E2: paragraphs 36–38). *“The conditions*,* like how quickly you know which days those are. […] That sometimes stresses me out and does not contribute positively to my mental well-being in that moment”* (E9: paragraph 26).

Spatial conditions such as uncomfortable seating or unattractive rooms are also described as additional burdens that have a negative impact on well-being in everyday student life.

*“Definitely negative*,* sitting at the desk and online lectures”* (E8: paragraph 30). *“I personally would appreciate it if the rooms were just brighter and nicer. Yes*,* you do not necessarily feel comfortable sitting there as a student”* (E15: paragraph 50). *“Unconsciously*,* I would think about things like the chairs*,* how the spatial facilities are set up. How many people do you sit with in a room”* (E3: paragraph 38).

**Fig. 1:**
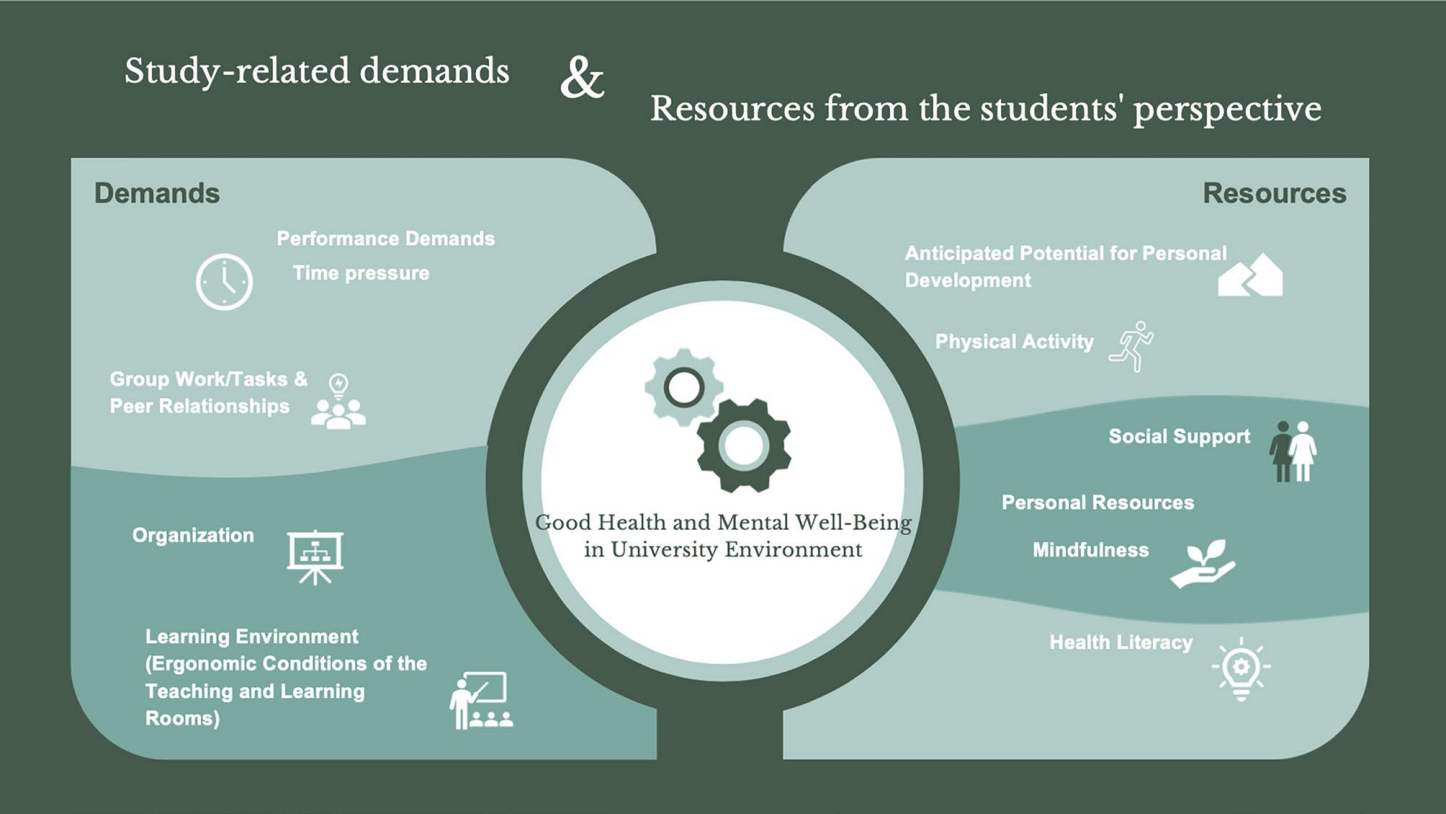
Results: study-related demands and resources (own illustration by Sara Kheiravi and INJ)

In summary, performance requirements, time pressure, group work, peer relationships, organizational conditions and ergonomic aspects of the learning environment (Fig. 1) are the most important study-related demands that affect students’ mental well-being. In the SD-R model, performance requirements, time pressure and peer relationships are regarded as health-damaging features of a degree programme that lead to mental discomfort and impair students’ health and performance [25].

### Study-related resources (Fig. 1)

#### Anticipated potential for personal development

Students do not perceive the study demands exclusively as a burden, but at the same time recognize them as potential resources for personal development and the promotion of their own health.

*“Additionally*,* the studies. Indeed*,* one of the reasons I decided to pursue it was to develop myself further. (…) To change*,* to learn something new again in the sense of becoming a bit fitter and gaining a fresh perspective mentally*,* receiving new stimuli*,* new ideas*,* acquiring new knowledge”* (E19: paragraph 32). *“Therefore*,* and I’m doing my master’s. Although it stresses me out as well*,* it is also a resource for me because I simply enjoy it*,* including the subject matter itself”* (E15: paragraph 42). *“And variety*,* meaning not stagnating*,* but constantly learning new things and being interested in new perspectives in life”* (E3: paragraph 32).

Examination formats, practical learning opportunities and social interactions are perceived as central fields of learning that contribute to the expansion of personal development.

*“The degree programme here is quite grateful. We have a lot of oral exams*,* and you can learn a lot about yourself during your studies” (E13: paragraph 34). “That always stressed me out a bit*,* but it also had its advantages in terms of simply adapting to different people and adjusting your pace”* (E4: paragraph 48).

In particular, dealing with the progression of illnesses and reflecting on the effects of unhealthy behavior promotes a critical examination and further development of one’s own understanding of health and illness. The degree programme is not only understood as a professional qualification process, but also as a phase of personal development and the assumption of personal responsibility for one’s own health.

*“So I also learn from the patients that I look after in some way*,* so to speak. Even if it’s just that I see what certain things or habits do to people. For example*,* smoking is the perfect example of a severe COPD Gold 4 patient who can no longer cope at all without their NIV ventilation”* (E14: paragraph 38).

#### Physical activity and social support

In dealing with study-related stress, students draw on various health-promoting resources. Physical activity in the form of regular exercise is described as a conscious strategy for coping with stress and relieving mental strain. Physical activity not only serves physical health but is also perceived as a means of finding inner peace and promoting mental balance.

*“I recently discovered jogging for myself*,* and it is a wonderful opportunity and way for me to relax a bit”* (E19: paragraph 30). *“Regular exercise*,* or I try to exercise regularly and interact with friends”* (E6: paragraph 28). *“I spend a lot of time outdoors. Yes*,* I talk a lot. Therefore*,* discussing things truly helps to maintain my health”* (E12: paragraph 28).

In addition, social interaction with other students is an important factor for emotional regulation. The open exchange about personal worries, fears and burdens enables students to put their challenges into perspective and experience emotional relief. The dialogue creates the awareness that they are not alone with their own problems, which is experienced as psychologically stabilizing. A supportive social study environment, characterized by cohesion and a shared ‘mood’, also contributes to social well-being.

*“(…) I am the kind of person who opens up when I’m not feeling well*,* when something is stressing me out*,* when something is not working*,* or when I’m afraid of something. I always find that once I have talked about it*,* everything seems half as bad*,* and I also realize*,* okay*,* others are in the same boat. Others might feel the same way”* (E9: paragraph 24). *“Yes*,* just like in my third semester*,* when I was worried about not passing the courses or feeling overwhelmed*,* that’s when talking about it helps”* (E18: paragraph 44). *“It’s also because the fellow students are truly excellent*,* and I think it is rare to find that—probably more common in these social study programs—where you’re on the same vibe with a large group”* (E13: paragraph 69).

### Personal resources

The students interviewed are characterized by a reflective and responsible approach to their own health. They have a pronounced self-awareness and ability to self-regulate, which enables them to recognize personal stress limits at an early stage and contribute to maintaining their health through targeted actions. They understand health as a process that can be actively shaped.

*„The ability to set boundaries and say no. Having a relatively good sense of when I am leaning toward health and when toward illness. In addition*,* being able to take the appropriate actions“* (E4: paragraph 40). *„Yes*,* simply listening to the body somehow. If it tells me I cannot go on*,* then I take a day off. No matter what*,* whether it is work*,* studies*,* or anything else“* (E11: paragraph 30). *“The ability to not let myself get too stressed*,* so that I remain very calm*,* I believe*,* is a good resource”* (E10: paragraph 29).

In addition, students have solid health-related skills that they have acquired as part of their vocational training. This knowledge includes both theoretical knowledge about health-promoting behavior and practical skills for implementation in everyday life. The training is seen as a resource that enables students to deal with health challenges competently and preventively.

*„The resource that through my profession I know what I need to do to stay healthy or to keep my health in check. Without the training*,* it might be different”* (E8: paragraph 26). *„In any case*,* yes*,* also through the training*,* I mean*,* you do know what’s good and not good for body and mind*,* so I think that is also a resource for me”* (E10: paragraph 29). *„(…) that I seek help when I need it*,* and now I also know where to go”* (E11: paragraph 28).

The resources identified in the study - such as the potential for personal development, physical activity, social support and personal resources (Fig. 1) - represent key factors that promote health in the context of studying. According to the SD-R model, potential for personal development and social support are understood as health-promoting features of the study context. They make a significant contribution to successfully mastering study-related challenges and have a positive effect on students’ health and performance [25].

### What attitudes and ideas do students perceive regarding the integration of health promotion into the curriculum?

Overall, students view the integration of health-related content into the curriculum as particularly effective and meaningful. They believe that embedding such learning formats in regular courses reaches more students and has a more lasting impact than stand-alone programmes or passive information channels like emails.

*“As part of*,* for example*,* health promotion*,* it is only sensible. For instance*,* I actually attend all lectures quite regularly*,* and therefore*,* I believe it is quite good. This way*,* you can definitely reach students much better than through email channels or something like that”* (E13: paragraph 85). *“However*,* I believe there is great interest in further promoting one’s own health*,* especially in these social professions. In addition*,* yes*,* that is why I consider these to be quite good approaches*,* like incorporating it into a module handbook*,* or into the modules themselves*,* because that way*,* people can be made aware of it”* (E14: paragraph 90). *“Well*,* I actually think that nursing students tend to neglect their own health. Both during their studies and in everyday life*,* in their profession. And that is actually something that should be much more in focus”* (E16: paragraph 59).

### Individual and voluntary

At the same time, students emphasize the importance of considering essential framework conditions: learning opportunities should be based on voluntary participation and tailored to individual needs. The heterogeneity of the student body – characterized by diverse professional backgrounds, age groups, and learning objectives – necessitates a differentiated and individualized design of health-related educational content.

*„Especially when considering that the age range in our program*,* for example*,* is quite broad*,* it is very sensible in terms of addressing various needs. For people who have just completed their training*,* those who have worked for eleven years*,* so to speak*,* or those with more experience and older age. I would find this reasonable*,* and it should be tailored to diverse needs“* (E2: paragraph 50). *“However*,* I think if this is truly done as an additional seminar*,* it may not benefit everyone - the content needs are different for everyone. I think it misses the target. In addition*,* I believe*,* while a lot can be clarified*,* ultimately everyone has very individual issues*,* and I think such a curriculum or course does not necessarily address the individual person”* (E4: paragraph 66).

Students highlight the significance of voluntary participation, alongside content relevance, as a key factor influencing the acceptance and effectiveness of health promotion programmes.

*“I think many people wouldn’t really engage with it and would rather feel annoyed because they would have to spend even more mandatory time on it. I believe it should rather be a voluntary offer*” (E5: paragraph 46). *“I think it would be difficult if every week we were told*,* ‘Now you have to do mindfulness training with us*,*’ or*,* ‘You have to do this or that.’ I feel like it should stay an optional offer “*(E15: paragraph 65).

### Mental health and wellbeing

Mental health emerged as a key topic in the interviews. Its importance was particularly highlighted in the context of the COVID-19 pandemic, during which students experienced increased psychological strain.

*“Owing to the coronavirus situation*,* I believe that mental health has not flourished exactly but rather suffered. That is why I think it is good to have a focus on it and to continue addressing it*,* rather than just briefly mentioning it and then dropping it”* (E9: paragraph 43). “*It makes a lot of sense*,* because I don’t think that all people are so attached and not all people take this step and say okay*,* so I think it’s more likely with physical problems. But I think it’s still a huge taboo when it comes to mental health problems and not everyone makes concessions and seeks help. And I think that an offer at the university might be a bit low-threshold*” (E11: paragraph 46).

The students attach relevance to the topic in relation to the nursing profession. They see the promotion of their own mental health as a job-related competence that is of central importance for activities in the healthcare sector. From their point of view, anchoring this in the curriculum could help them to develop a health-promoting attitude that benefits both their own well-being and their professional competence. The students believe that promoting mental health plays a key role in preventing university dropouts and fostering long-term job satisfaction.

“*I think that makes a lot of sense. You have to say*,* especially now in the nursing field. It’s an occupational field where you have a lot of dropouts and where I think we also have a lot of university dropouts. I think so*,* but I don’t know. And I think that often has something to do with health. So*,* of a mental nature*,* but certainly also of a physical nature. And if you dedicate a module to the whole thing*,* where you create a space for it*,* then I think that’s a good thing*” (E16: paragraph 52). *“[…] What I think is important is to really raise awareness about this. It should be properly integrated into the curriculum. What impact does working in this system have on us? What challenges might we face? Things like the cool-down effect*,* burnout*,* and overload. And maybe you should already learn during your studies what the warning signs are and what can go wrong*” (E15: paragraph 65).

Summary, students see the integration of health promotion into the curriculum as an opportunity as well as a challenge. In their view, the balance between structural integration and individual time flexibility is important. In their opinion, strategic embedding could improve health awareness, self-care and resilience for prospective and postgraduate health professionals.

## Discussion

We presented the results of interviews conducted with students from the Department of Nursing & Management at a University of Applied Sciences in Hamburg, Germany. The aim of the study was to examine which demands of studying are perceived as stressful by the students and how these affect their subjective health. We were also interested in what resources students use to cope with the demands and how they define health. Furthermore, we examined students’ attitudes towards the integration of health promotion into the curriculum. In the following section, our findings are discussed in the context of national and international findings.

### Students understanding of health

The students’ perceptions of health align with the theory of subjective health [47], which highlights the significance of individual experiences and personal lifestyles in shaping how health is understood [47]. This theoretical perspective helps to contextualize the students’ holistic and resource-oriented views, in which emotional well-being, social relationships, and everyday life experiences are central [47, 48]. This has important implications for intervention design, as subjective health concepts and individual needs are key to ensuring effectiveness [48]. These findings are consistent with the WHO’s definition of health as ‘a state of complete physical, mental and social well-being and not merely the absence of disease or infirmity’ [49]. However, this definition has been criticized for not adequately reflecting contemporary life realities, such as living with chronic illness or the challenges of aging [50]. As a response, scholars have proposed a dynamic understanding of health that emphasizes adaptability and self-management in everyday life [51]. The students’ perspectives reflect this shift, viewing health as a continuous, dynamic process encompassing physical, psychological, and social dimensions. This pluralistic view accommodates diverse individual definitions and recognizes the complexity of personal health experiences [52]. Huber et al. [50] capture this understanding by defining health as ‘the ability to adapt and to self-manage in the face of social, physical, and emotional challenges’ [50].

### Study-related demands and health

#### Academic demands as core stressors

From the perspective of our students, academic performance and time pressure constitute key study-related demands that significantly contribute to the development of stress, thereby negatively affecting mental health and overall well-being. This experience of strain is consistent with the study demands-resources framework [25], which illustrates the dynamic interplay between academic demands and available resources. A representative survey conducted at Charité – Universitätsmedizin Berlin, Germany identified time-related demands as the most pressing issue from the students’ perspective [34]. Conversely, high satisfaction with academic demands was found to be a significant predictor of lower psychological distress [34]. International studies also confirm that high academic workloads and examinations are associated with increased subjective stress and impairments in students’ mental health [53–55]. Our participants reported experiencing particular stress when academic responsibilities had to be managed concurrently with personal obligations. However, a study in UK found limited evidence in their study that nursing students experience significant stress due to the reconciliation of academic and private responsibilities, or that they struggle to balance study and personal life [54]. Similarly, in our study, several students perceived study-related demands as less burdensome. This aligns with transactional stress theory, which posits that objective demands alone are insufficient to explain stress responses—subjective appraisal is crucial [56]. Students who perceive academic challenges as opportunities for growth tend to demonstrate greater resilience and better stress management [57]. In our study, some students described their studies as a positive opportunity for personal development—an experience associated with higher life satisfaction and better health outcomes [58]. Within the SDR framework, the anticipated qualification potential is conceptualized as a health-promoting resource [25]. However, cross-national comparative studies show a consistent pattern of core stressors [35]. A survey of 110 nursing students in Ireland found that the clinical learning environment, academic pressure and interpersonal relationships were key sources of stress [59]. Similar findings are reported by Burnard et al. [55] across five nations and by Pulido‑Martos et al. [35] in their global review. Universities constitute essential living environments for students and therefore have significant leverage in the prevention of study-related stress [18, 19]. At the structural level, examination formats and submission deadlines should be designed to allow for greater flexibility and to prevent peak stress periods [15, 34]. Program-based interventions focused on time and self-management may serve as effective strategies for mitigating time-related stress [58, 60]. Such interventions explicitly align with the expressed needs of students [61]. In addition, low-threshold opportunities for social interaction can foster a sense of belonging [53] and reduce students perceived stress levels [28, 30].

#### Group work and peer relationship

Stressors affecting the mental health of our interviewees included group task and interpersonal challenges. The literature indicates that cooperative collaboration in groups often proves difficulties, as disagreements, a lack of direction, and an absence of goal orientation can occur [62]. Establishing binding collaborative rules has been found to be an effective condition to minimize those difficulties [62]. Furthermore, psychosocial stressors, which arise not only in group work, are perceived as burdensome by many people regardless of individual assessment processes [63]. In their review of qualitative studies, Hurst et al. [64] identified interpersonal conflicts and relationships as the most common stressors among students [64]. These relational stressors affect both private and academic contexts and can negatively impact overall well-being [64]. This finding is relevant within the university setting, as students primarily form contacts and relationships with their peers, which can amplify the effects of these stressors [65]. Data from Baik et al. [61] suggest that students desire more understanding from their peers and educators regarding their diverse life situations, such as balancing family and studies [61]. Thus, fostering a generally positive atmosphere among students could help reduce stress and promote well-being [58, 61].

#### Organization and learning environment

Our students perceive poor organization of study conditions and a lack of support and communication from lecturers as significant risks to their well-being. This finding in line with those of other studies indicating that a lack of contact [64], poor communication [61], and insufficient understanding from professors negatively impact student well-being [61, 65]. A study indicates that students receive the least support from teaching staff [66]. In contrast, Hurst et al. [64] report that insufficient support from peers also represents a significant source of stress [64]. This underscores the importance of a supportive environment [61, 67]. According to Baik et al. [67], a supportive and appreciative environment, along with stable interpersonal relationships within the university context, plays a critical role in promoting student mental health. These findings underscore the broader importance of fostering a socially supportive academic environment [61, 67]. Furthermore, Aloia and McTigue [68] show that informative and emotionally supportive communication from family, friends, and university staff can help safeguard students’ mental well-being—especially among those who perceive their workload as particularly high [68].

#### A lack of ergonomic workplace

Design, such as inadequate lighting, is rated by our students as a demand that is a risk to health. This result is in line with national [65] and international findings on students in general [61]. Specifically, prolonged periods of sitting by students have a negative effect on their physical and mental health [69]. Baik et al. [61] reported that students express a desire for ergonomically adequate seating and workspaces [61]. However, there is a lack of ergonomic furniture and measures to reduce sitting time in universities [69]. Recommended measures include the provision of ergonomic furniture, height-adjustable standing desks, or opportunities for active interruptions of sitting [69]. Taking legal occupational safety regulations into account can help design the learning environment ergonomically [70].

### Study-related resources and health

#### Anticipated potential for personal development

The Study Demands-Resources Framework assumes that study demands and study resources interact with students’ engagement and health. ‘Anticipated qualification potential’ can be understood as a study resource—more specifically, as a positive expectation regarding academic outcomes or perceived competence gain. According to the model, study resources promote student engagement and have a positive effect on mental health [25]. In this context, the findings of Zimmermann et al. [6] are particularly relevant: students were motivated by the personal quality gains associated with higher education, the societal recognition of academic nursing education, and the prospect of diverse career opportunities [6]. These motivational factors reflect the expected development potential, which can be interpreted as an important personal resource for the students.

#### Physical activity

For our students, physical activity and movement serve as key resources in coping with the demands of academic study. Other studies have also demonstrated that engaging in physical exercise is crucial for managing stress and burden among students [32, 71]. Recent data from a national student survey indicate that physical activity and social contacts are the most commonly used stress-compensating strategies [15]. Therefore, sports and physical activity opportunities should be more thoroughly integrated into daily academic life, actively promoted by universities, and considered in curriculum design [71].

#### Social support and personal resources

According to our participants, social interaction, self-care, and health literacy serve as valuable resources for managing academic demands. These findings align with other studies that also highlight that peers and family are important resources for coping with study demands and personal challenges [32, 61, 66]. Studies show that social support is an important resource for students’ mental well-being and resilience [27, 28, 58, 72]. Moreover, our students appear to demonstrate a relatively high level of health literacy as a personal resource, which they attribute to their prior vocational training and practical experience in the healthcare sector. Health literacy is considered a key health-promoting resource that enables individuals to make informed and health-conscious decisions [73]. This is particularly important given the demanding nature of the academic nursing programme and the challenges associated with clinical setting [1, 35, 36, 38]. However, existing national research indicates that students enrolled in health-related degree programmes often exhibit comparatively low levels of health literacy, highlighting the need for targeted support and development in this area [74, 75].

#### Curriculum and health promotion

Our students express a strong interest in integrating health-related modules into the academic curriculum. They perceive a particular need in the area of student mental health, especially in the context of their health-related degree programs. At the same time, they emphasized that access to such offerings should remain voluntary and that individual approaches are necessary to accommodate diverse needs. The aspects raised by the students are consistent with the findings of Baik et al. [61] and Upsher et al. [76], who also report that the integration of mental health into university studies is welcomed by students [61, 76]. In relation to mental health, the identification of individual needs is highly important, and it is important to recognize students with specific needs in this area [67, 77].

#### Policy implications and future research

In international comparison, the study‑related stressors perceived by our students broadly mirror those reported by nursing students elsewhere [35, 54, 55]. Our findings, however, stem from a small qualitative study conducted at a single German university. Further research is therefore warranted. First, a nationwide, representative investigation is needed to objectively assess the structural conditions of nursing degree programmes under the 2020 Nursing Professions Act [1] and their influence on students’ health. The Bielefeld Questionnaire on Study Conditions and Health [78] could serve this purpose, complemented by instruments that capture stress arising from both academic and clinical settings [36, 55], such as the Stress in Nurse Education Questionnaire (SINE). Second, the evidence generated should underpin a policy brief for German decision‑makers, stimulating further debate on the framework and demands of nursing education and informing subsequent measures. The recent introduction of a study allowance through the Act to Strengthen Nursing Degree Programmes “Pflegestudiumstärkungsgesetz” [79] demonstrates the legislature’s readiness to enact structural changes that enhance the attractiveness of nursing studies.

### Strengths and limitations

The strengths of this study include addressing an important topic: study-related demands and health of students in nursing and healthcare professions. The interview data was analyzed close to the participants’ original words, making their viewpoints clear and easy to understand. The qualitative descriptive method avoids complicated theories and keeps assumptions simple. This approach offers an open and unbiased look at a topic that has not been widely studied, allowing important connections to be identified. The findings have practical relevance, as they can help universities improve study conditions and better support student health. Additionally, the study benefited from easy access to participants. Because of close connections with the university department, suitable interviewees were quickly found, trust was built, and detailed personal insights were gained.

However, we would like to acknowledge the limitations of our study. A key limitation of the qualitative descriptive method is its primary focus on presenting phenomena as described by participants, without engaging in comprehensive interpretation or critical analysis. Consequently, the findings remain largely descriptive and may not uncover complex relationships or deeper insights. Additionally, our sample consisted of students with a health background, which may have resulted in heightened health awareness—or even over-sensitization—potentially leading them to perceive certain demands as less stressful. As the target population comprised students from a single university in Germany, caution should be exercised when generalizing the results beyond this specific context. Furthermore, the common issue of self-selection bias applies, as participants typically exhibit a willingness to reflect and engage in dialogue. This may introduce socially desirable response behavior, which could affect the validity of the results. As with all qualitative research, the analysis may be influenced by the subjective interpretation of the researchers. To enhance the trustworthiness of the findings, strategies such as reflexive journaling were employed. Nevertheless, complete objectivity cannot be expected in this context.

## Conclusion

In summary, this study identified study-related stress factors and their impact on mental well-being from the students’ perspective. The stressors reported by our students largely correspond to those identified in international studies involving nursing students in other countries. The current study is limited by the small sample. However, the findings highlight the importance to create healthy study conditions. Findings are particularly relevant for universities and educators in Germany, who would like to become more aware of the study-related stressors of nursing students and discuss suitable preventive measures. Further quantitative research should examine the academic demands and their impact on students’ mental health. A nationwide, representative study would be a valuable next step.

## Supplementary Information

Below is the link to the electronic supplementary material.

Supplementary Material 1

## Data Availability

The raw data used in this study are available from the first author (Ivonne-Nadine Jürgensen) upon reasonable request.
