# Supplementary material for: Study-related demands, resources and curriculum from students’ perspective: results of a qualitative descriptive study among students in nursing and healthcare in Germany
Source: BMC Nurs. 2025 Aug 11;24:1054. doi: 10.1186/s12912-025-03692-8 (PMC12341071; doi:10.1186/s12912-025-03692-8)
Supplement: Supplementary file 1 — Supplementary Material 1 [file 12912_2025_3692_MOESM1_ESM.pdf]

Appendix 1

Qualitative interviews with students to explore their understanding of health, study demands and resources

General information about the interview:

1) Inform and clarify the interviewee about the study
2) Give information letter and consent form
3) Sign consent form for the study
4) Start the interview

Interview guide*

**Socio-demographic data**
Age in years:
Gender: m / f / d
Marital status: single / in relationship / married / divorced / widowed
Vocational training (healthcare sector):
Years of professional experience (in the healthcare sector):
Part-time job during your studies?<br>If yes, how many hours per week:
Financial support? Yes / No
**Initial question health**
• What does health mean for you personally?
**Main part (note: all questions relate to physical and mental health)**
• When you look at your life, what role does your own personal health play?<br>• What factors influence your health? Which ones are most important for your health?<br>• Has the importance of health changed during your studies? If so, please tell me how?<br>• What are your resources that promote and maintain your health?<br>• What do you do to maintain and promote your health?
• What general conditions and challenges of your study programme affect your health?<br>• Are there any circumstances in the department that affect your health?<br>• Have you ever thought about dropping out of your study programme? If so, when and why?
• Which university health promotion programmes are you aware of?<br>• What health promotion programmes do you use?<br>• If the Department of Nursing & Management were to design health promotion programmes, which ones would you personally wish for?<br>• What would it take for you to take part in these programmes?<br>• To what extent would you consider it useful to implement the offers in the curricula of the degree programmes?
**Finish**
• Is there anything else you would like to add on the subject?

*Translated from German language to English language using DeepL (www.DeepL.com/Translator) (free version)
